# Hepatitis B Virus Polymerase Suppresses NF-κB Signaling by Inhibiting the Activity of IKKs via Interaction with Hsp90β

**DOI:** 10.1371/journal.pone.0091658

**Published:** 2014-03-11

**Authors:** Dan Liu, An’dong Wu, Lei Cui, Ruidong Hao, Yuan Wang, Jing He, Deyin Guo

**Affiliations:** National Key Laboratory of Virology and Modern Virology Research Center, College of Life Sciences, Wuhan University, Wuhan City, P. R. China; Academia Sinica & National Defense Medical Center, Taiwan

## Abstract

Nuclear factor-κB (NF-κB) plays a central role in the regulation of diverse biological processes, including immune responses, development, cell growth, and cell survival. To establish persistent infection, many viruses have evolved strategies to evade the host’s antiviral immune defenses. In the case of hepatitis B virus (HBV), which can cause chronic infection in the liver, immune evasion strategies used by the virus are not fully understood. It has recently been reported that the polymerase of HBV (Pol) inhibits interferon-β (IFN-β) activity by disrupting the interaction between IKKε and the DDX3. In the current study, we found that HBV Pol suppressed NF-κB signaling, which can also contribute to IFN-β production. HBV Pol did not alter the level of NF-κB expression, but it prevented NF-κB subunits involved in both the canonical and non-canonical NF-κB pathways from entering the nucleus. Further experiments demonstrated that HBV Pol preferentially suppressed the activity of the IκB kinase (IKK) complex by disrupting the association of IKK/NEMO with Cdc37/Hsp90, which is critical for the assembly of the IKK complex and recruitment of the IKK complex to the tumor necrosis factor type 1 receptor (TNF-R1). Furthermore, we found that HBV Pol inhibited the NF-κB-mediated transcription of target genes. Taken together, it is suggested that HBV Pol could counteract host innate immune responses by interfering with two distinct signaling pathways required for IFN-β activation. Our studies therefore shed light on a potential therapeutic target for persistent infection with HBV.

## Introduction

It is estimated that more than 350 million individuals are chronically infected with hepatitis B virus (HBV), nearly a quarter of whom will eventually develop severe liver diseases, including liver cirrhosis and hepatocellular carcinoma (HCC), the latter being one of the most common forms of human cancer [1]. HBV is an enveloped DNA virus that belongs to the Hepadnaviridae family. It contains a small genome (approximately 3.2 kb), composed of a partially double-stranded (DS) relaxed-circular (RC) DNA structure that replicates by reverse transcription via an RNA intermediate, the pregenomic RNA (pgRNA) [2]. The HBV polymerase (Pol) is a multifunctional protein that consists of the following four domains: terminal protein (TP), reverse transcriptase (RT), RNaseH, and a non-conserved spacer domain between the TP and RT domains. A unique feature of hepadnavirus reverse transcription is the Pol (reverse transcriptase)-primed initiation of minus-strand DNA synthesis, which requires the recognition and binding of Pol to a stem-loop structure, called epsilon (ε), located at the 5′ end of the pgRNA [3], [4]. It has been demonstrated that heat shock protein 90β (Hsp90β), in a dynamic process that is dependent on ATP hydrolysis, interacts with HBV Pol and facilitates Pol-ε interaction in HBV [5]–[7].

Hsp90 is an abundant, highly conserved cellular chaperone that functions as a key component of a multiprotein chaperone complex, which includes Cdc37 and several other proteins that regulate folding, maturation, stabilization, and renaturation of a select group of target proteins [8], [9]. It has been shown that Hsp90 interacts with IκB kinases and signaling proteins of the nuclear factor-κB (NF-κB) pathway, including MEKK3, NIK, RIP1, TAK1, and TBK1 [10]–[14], and Hsp90-Cdc37 serves as a transiently acting essential regulatory component of the IκB kinase (IKK) signaling [15].

NF-κB family members play crucial roles in the regulation of genes involved in diverse biological phenomena, such as inflammation, immune responses, carcinogenesis and apoptosis [16]. The family includes five members, NF-κB1 (p105 and p50), NF-κB2 (p100 and p52), RelA (p65), RelB, and c-Rel, all of which share a highly conserved N-terminal Rel homology domain responsible for DNA binding, homo- or hetero-dimerization, and nuclear translocation [17]. The NF-κB activation pathways can be classified into canonical and non-canonical pathways; the canonical pathway leads to the degradation of IκB, whereas the non-canonical pathway involves the processing of p100 to the mature subunit, p52 [18]. In the canonical pathway, a broad range of extracellular stimuli, including bacterial or viral pathogens, antigens, mitogens, and inflammatory cytokines, induce diverse intracellular cascades that activate IKK complex composed of two catalytic subunits (IKKα and IKKβ) and a regulatory subunit (IKKγ or NF-κB essential modulator [NEMO]) [19]. Kinases that phosphorylate IκBα have been identified to compose a high molecular weight IKK complex whose catalytic activity is generally carried out by three tightly associated IKK subunits. IKKα and IKKβ serve as the catalytic subunits of the kinase, and IKKγ serves as the regulatory subunit; activation of IKK depends upon its phosphorylation. Ser177 and Ser181 in the activation loop of IKKβ (serine 176 and 180 in IKKα) are specific sites that, when phosphorylated, cause conformational changes, resulting in kinase activation [20], [21]. IKK-mediated phosphorylation triggers IκB and p105 polyubiquitination by the SCF^βTγCP^ E3 ligase complex and subsequent proteasomal degradation, resulting in the release of p50-, p65-, and c-Rel-containing heterodimers and translocation into the nucleus to regulate gene transcription [22]. The non-canonical pathway is activated by a subset of receptors on B cells, such as CD40 and B-cell activating factor (BAFF-R) [23]–[26]. These receptors initiate a signaling cascade, leading to activation of IKKα, which phosphorylates p100. Phosphorylated p100 is polyubiquitinated, and its C-terminus is then selectively degraded by the proteasome, sparing the N-terminal Rel homology domain, which goes on to generate the mature p52 subunit. p52 forms a dimer with RelB, and the dimeric complex enters the nucleus to induce the expression of downstream genes. Through the two pathways described above, NF-κB controls gene expression, including genes encoding proinflammatory cytokines (e.g., interleukin 1[IL-1], IL-2, IL-6, IL-8, TNF-α, BAFF, and BLyS), chemokines (e.g., IL-8, MIP-1α, MCP1, RANTES, eotaxin, B-lymphocyte chemoattractant, and secondary lymphoid tissue chemokine), adhesion molecules (e.g., intercellular adhesion molecule 1, vascular cell adhesion molecule 1, and E-selection), inducible enzymes (e.g., COX-2 and inducible nitric oxide synthase), growth factors, some acute-phase proteins, and immune receptors, all of which play critical roles in controlling inflammatory processes [27], [28].

Most research on HBV Pol concerns its polymerase function in the viral life cycle, its potential immune evasion mechanisms are rarely addressed. Recently, a report has shown that HBV Pol inhibits interferon-β (IFN-β) induction by disrupting the interaction between IκB kinase-ε (IKKε) and the DEAD box RNA helicase (DDX3) [29]. In the current study, we found that HBV Pol suppressed the activity of the IKK complex by preferentially disrupting the association of IKK/NEMO with Cdc37/Hsp90, which is critical for the assembly of the IKK complex and recruitment of the IKK complex to the tumor necrosis factor type 1 receptor (TNF-R1). We also demonstrated that HBV Pol inhibited NF-κB activity and the production of key downstream inflammatory molecules. Consistent with accumulating evidence, it is possible that HBV is involved in regulating the innate and adaptive immune responses of its host by interfering with two distinct pathways. Our study helps define HBV Pol as a vital protein that can counteract immune responses and contribute to persistent HBV infection.

## Experimental Procedures

### Construction of Expression Plasmids

The cDNA encoding the HBV polymerase was amplified by PCR from pT-HBV1.3, which contains the full length HBV genome and a 0.3-fold redundant fragment (Guangxia Gao, Institute of Biophysics, Chinese Academy of Sciences), using the forward primer, 5′-GCG TCG ACA CCA TGC CCC TAT CCT ATC AAC ACT T-3′, and the reverse primer, 5′-ATA AGA ATG CGG CCG CTC ACG GTG GTC TCC ATG CGA-3′. The resulting PCR product was then cloned into pAHC, which was reconstructed from pCI-Neo (Promega) with an ATG-HA-tag at the N terminus (a gift from Prof. T. Mäkelä, University of Helsinki, Finland) using standard cloning methods, resulting in pAHC-HA-Pol. The HBV X and Core proteins were also inserted into the pAHC vector using the same procedure. The recombinant plasmids were analyzed by restriction digestion and PCR, and the sequences were verified by sequencing. The fragment of Hsp90β was amplified by PCR from cDNA that was reverse transcribed from the extracted total RNA from 293 T cells with the forward primer, 5′-CCC GAT ATC AAT GCC TGA GGA AGT GCA C-3′, and the reverse primer, 5′-ACG CGT CGA CCT AAT CGA CTT CTT CCA TGC-3′, and cloned into the pCMV-tag-2B vector as described above, resulting in the pCMV-flag-Hsp90β construct. The expression plasmids, IKKα, IKKβ, TRAF2, and TRAF6, and the luciferase reporter plasmids, p5×NF-κB-luc and pIFNβ-luc, were kindly provided by Dr. Hongbing Shu (College of Life Science, Wuhan University). The expression plasmids, pcDNA3.1(+)-NIK and dominant-negative NIK [pcDNA3.1(+)-dnNIK], which has the kinase-dead mutation, K429A/K430A, were kindly provided by Brian M. J. Foxwell and Alison Davis (Kennedy Institute of Rheumatology Division, Imperial College School of Medicine, London, United Kingdom). The pGL3 IL-6-p-Luc plasmid and its mutant, pGL3 IL-6-p-Luc (ΔNF-κB), and the pGL3 IL-8-p-Luc plasmid and its mutant, pGL3 IL-8-p-Luc (ΔNF-κB), were a gift from Ying Zhu (State Key Laboratory of Virology, College of Life Science, Wuhan University). The HBV replicon and its three mutants are described in the supplementary information.

### Cell Lines and Cell Culture

The human hepatoma cell lines, wild-type HepG2 and Huh7, were obtained from the China Center for Typical Culture Collection (CCTCC) (Wuhan, China). The stable HBV-producing cell line HepG2.2.15 (ayw subtype) was kindly provided by Dr. Ying Zhu from Wuhan University, HEK 293 T cell line was kindly provided by Dr. Hongbing Shu from Wuhan University. All cell lines mentioned above were cultured in 90% Dulbecco’s modified Eagle’s medium (Gibco) containing 10% heat-inactivated fetal bovine serum (Gibco), 100 µg/ml streptomycin sulfate and 100 U/ml penicillin G sodium at 37°C and 5% CO_2_ in an incubator.

### Plasmid Transfection; TNF-α, LPS, MG-132, and Geldanamycin Treatment; and Luciferase Reporter Assays

DNA transfection was performed using the FuGENE HD Transfection Reagent (Roche) according to the manufacturer’s instructions; each transfection was normalized using corresponding vector plasmids. At 24 h posttransfection, the cells were harvested and treated with TNF-α (10 ng/ml; Sigma), LPS (1 µg/ml; Sigma), or MG-132 (20 µmol/liter; Alexis). For treatment with Geldanamycin (GA), the cells were incubated with 10 µM Geldanamycin (Sigma) or with 0.1% DMSO vehicle for the indicated times. For the luciferase assay, cells were cotransfected with plasmids indicated in the figures, luciferase reporter vectors, and the Renilla luciferase reporter, pTK-Renilla-Luc (Promega, Madison, WI), which served as an internal control for transfection efficiency. Luciferase activity was measured by the Dual-luciferase assay system (Promega) according to the protocol recommended by the manufacturer. The luciferase assay was performed in triplicate, and the results are shown as means ± standard error (SE).

### Preparation of Cytoplasmic and Nuclear Fractions

At 36 h posttransfection, treated or untreated cells were washed three times with ice-cold phosphate-buffered saline and hypotonic buffer (10 mM HEPES [pH 7.9], 5 mM KCl, 1.5 mM MgCl_2_, 1 mM NaF, and 1 mM Na_3_VO_4_) supplemented with dithiothreitol (1 mM), phenylmethylsulfonyl fluoride (1 mM), aprotinin (2 µg/ml), leupeptin (2 µg/ml), and soybean trypsin inhibitor (37.5 µg/ml). After allowing for cell lysis by incubating for 15 min on ice, the cytoplasmic fraction was prepared by centrifugation at 3,000 rpm for 5 min and then cleared by another centrifugation step at 12,000 rpm for 15 min. Following these initial centrifugation steps, the pellet was washed three times with hypotonic buffer and suspended in high-salt buffer (10 mM HEPES [pH 7.9], 1.5 mM MgCl_2_, 0.2 mM EDTA [pH 8.0], 420 mM NaCl, 25% [vol/vol] glycerol, 50 mM β-glycerophosphate, 1 mM NaF, and 1 mM Na_3_VO_4_) supplemented with dithiothreitol (1 mM), phenylmethylsulfonyl fluoride (1 mM), aprotinin (2 µg/ml), leupeptin (2 µg/ml), and soybean trypsin inhibitor (37.5 µg/ml). The suspended cell pellet was incubated for 30 min on ice with occasional vortexing, and the nuclear fraction was collected after centrifugation at 12,000 rpm for 10 min.

### Western Blotting and Immunoprecipitation

The cell lysate was washed with cold phosphate buffered saline and incubated in lysis buffer (50 mM Tris-HCl [pH 7.4], 150 mM NaCl, 0.1% SDS, 1% NP-40, 1% Triton X-100, and 1 mM EDTA) containing a cocktail of protease inhibitors (Calbiochem) on ice for 30 min. After centrifugation at 13,200 rpm for 10 min, the supernatants were isolated, and the protein concentration was determined. Proteins were then separated by sodium dodecyl sulfate polyacrylamide gel electrophoresis (SDS-PAGE) and electrotransferred onto nitrocellulose membranes with a mini Trans-blot cell (Bio-Rad). The specific immunoreactive proteins were detected by enhanced chemiluminescence (Millipore) and exposure of X-ray film (FUJIFILM). For the immunoprecipitation assay, total cell lysate was prepared with immunoprecipitation assay cell lysis buffer (20 mM Tris-HCl [pH 7.5], 150 mM NaCl, 1 mM EDTA, and 1% Triton X-100) supplemented with the cocktail of protease inhibitors indicated above. Three to five micrograms of appropriate antibodies was added to the precleared cell lysate and incubated overnight at 4°C. Immune complexes were captured with 50 µl of protein A-Sepharose (Santa Cruz Biotechnology) for 2 h at 4°C and washed three times with washing buffer (immunoprecipitation assay lysis buffer supplemented with 0.5 M NaCl). The antibodies used in this study were as follows: anti-β-actin, anti-GAPDH, anti-cyclin T1, anti-p65, anti-p50, anti-IκBα, anti-p100/p52, anti-Hsp90α/β, anti-NEMO, anti-IKKα, anti-IKKβ, and anti-cdc37 (all from Santa Cruz Biotechnology); anti-Flag and anti-HA (Sigma); and anti-RelB, anti-phospho-IKKα (Ser 176/180)/IKKβ (Ser 177/181), and anti-phospho-IκB-α (Ser 32/36) (Cell Signaling).

### *In vitro* Kinase Assay

An *in vitro* kinase assay was performed using immunoprecipitated proteins and the N-terminal GST-tagged recombinant human IκBα (1–54), in 20 µl of kinase buffer containing 20 mM Tris-HCl (pH 7.6), 10 mM MgCl_2_, 0.5 mM DTT, 100 µM ATP, and 5 µCi of [γ-^32^P] ATP at room temperature for 30 min. Samples were analyzed by 10% SDS/PAGE and autoradiography.

### Quantitative RT-PCR Analysis

Total RNA was extracted using the TRIzol reagent (Invitrogen) and reverse-transcribed with the Reverse Transcription System (TaKaRa). Subsequently, the cDNA was used as templates for PCR to amplify the GAPDH, IL-6, or IL-8 genes using the following primers: the sense primer, 5′-CCA CCC ATG GCA AAT TCC ATG GCA-3′, and the antisense primer, 5′-TCT AGA CGG CAG GTC AGG TCC ACC-3′, for GAPDH; the forward primer, 5′-AAA CAA CCT GAA CCT TCC-3′, and the antisense primer, 5′-CAG GGG TGG TTA TTG C-3′, for IL-6; and the sense primer, 5′-AAG AAC TTA GAT GTC AGT GC-3′, and the antisense primer, 5′-ACT TCT CCA CAA CCC T-3′, for IL-8. The PCR conditions included denaturation at 94°C, annealing at 56°C, and extension at 72°C for 25 cycles using γTaq (TaKaRa).

### Measurement of IL-6 and IL-8 in Cultured Cell Supernatants

Twenty-four hours after transfection, HepG2 cells were stimulated with LPS. IL-6 and IL-8 levels in the culture supernatants of HepG2 cells were measured using enzyme-linked immunosorbent assay (ELISA) kits (Neobioscience) according to the manufacturer’s instructions.

### Real-time PCR Quantification of Intracellular HBV DNA

Total intracellular DNA was extracted from HepG2 cells transfected with indicated plasmids by a Genomic DNA isolation kit (tiangen biotech co., Ltd). Intracellular HBV DNA was quantified by Real-time PCR in a ABI 7300 system using 20 microgram as template. Forward and reverse primers were 5′-CTCGTGGTGGACTTCTCTC-3′ (2RC/CCS nt 256–274) and 5′-CTGCAGGATGAAGAGGAA-3′ (2RC/CCAS nt 421–404) as reported previously [30]. The relative HBV DNA levels were normalized to beta-globin levels and compared with a relative threshold method.

### Statistical Analysis

The results in our study were expressed as the means ± SE of experiments performed in triplicate. Statistical analysis was performed using the Statistical Package Social Sciences (SPSS) program, and significant differences among groups were determined by least significant difference analysis. The accepted level of statistical significance was determined to have a p-value of <0.05.

## Results

### HBV Pol Suppresses NF-κB Activity in a Dose-dependent Manner in different Cell Types

**Figure 1:**
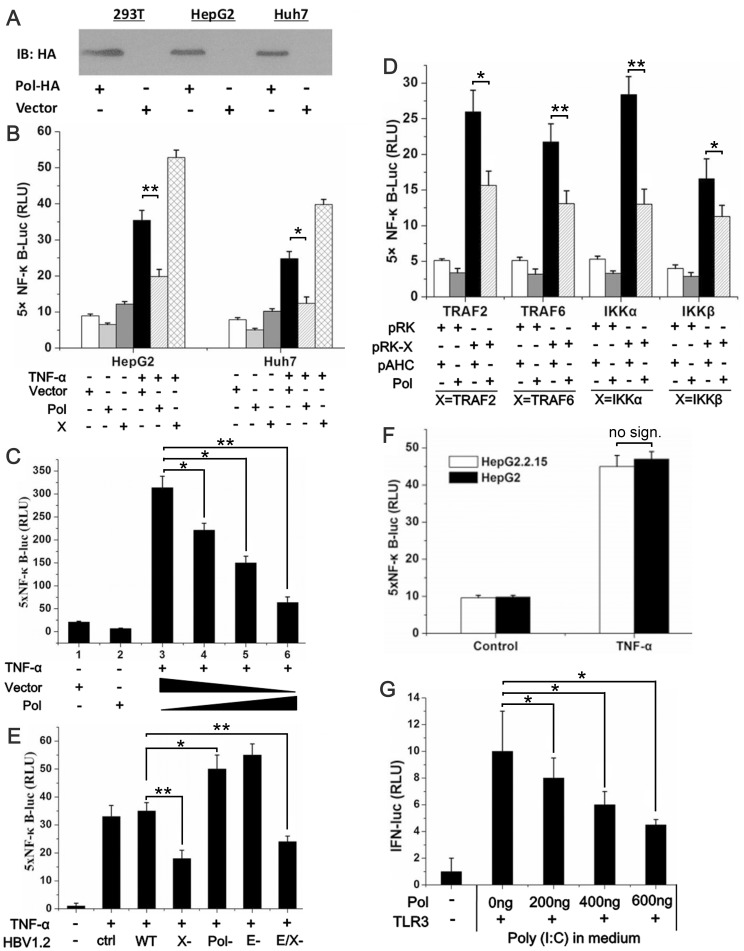
HBV Pol suppresses NF-κB activity in HEK 293 T, HepG2, and Huh7 cells. *A*. Western blot analysis revealed the expression of the HBV Pol in HEK 293 T (lanes 1 and 2), HepG2 (lanes 3 and 4), and Huh7 (lanes 5 and 6) cells. Cells were transfected with 0.8 µg of pAHC-Pol expressing the HBV Pol (lanes 1, 3, and 5) or 0.8 µg of empty vector (pAHC) as a negative control (lanes 2, 4, and 6). At 32 h posttransfection, expression of the plasmids was determined by western blot using a mouse anti-HA antibody. *B*. HepG2 and Huh7 cells were cotransfected with 0.1 µg of pNF-κB-Luc (5×NF-κB binding site promoter-driven luciferase reporter plasmid) and 10 ng of pTK-Renilla-Luc along with 0.6 µg of pAHC-Pol or 0.6 µg of pAHC-X as a positive control and normalized to the pAHC empty vector. At 24 h posttransfection, cells were treated with TNF-α (10 ng/ml) or left untreated for 1 h as indicated and then harvested for analysis by luciferase assay. *C*. 293 T cells were cotransfected with 0.1 µg of 5×pNF-κB-Luc and 25 µg of pTK-Renilla-Luc along with different amounts of pAHC-Pol (lane 3, 0 ng; lane 4, 200 ng; lane 5, 400 ng; lane 6, 600 ng). The total amount of plasmid was adjusted with the empty vector (pAHC) to 600 ng. At 24 h posttransfection, cells were treated with TNF-α (10 ng/ml) or left untreated for 1 h as indicated and then harvested for analysis by luciferase assay. *D*. HepG2 cells were cotransfected with 100 ng of 5×pNF-κB-Luc and 25 ng of pTK-Renilla-Luc together with 100 ng of the activating plasmid, pRK-X (where X is TRAF2, TRAF6, IKKα, or IKKβ), or the corresponding empty vector, pRK, along with 600 ng of pAHC-Pol or 600 ng of the pAHC empty vector as indicated below the graph. After 24 h of transfection, relative luciferase activities were determined. *E*. HepG2 cells were transfected with empty vector, WT, X-null, E-null, E/X-null and P-null HBV replicon together with the 5×pNF-κB-Luc reporter construct and pTK-Renilla-Luc, cells were treated with TNF-α (10 ng/ml) or left untreated for 1 h as indicated and then harvested for analysis by luciferase assay. *F*. HepG2.2.15 and HepG2 cells were transfected with 100 ng of 5×pNF-κB-Luc and 25 ng of pTK-Renilla-Luc. At 24 h posttransfection, cells were treated with TNF-α (10 ng/ml) or left untreated for 1 h as indicated and then harvested for analysis by luciferase assay. *G*. HepG2 cells were transfected with increasing doses of the HBV Pol expression construct, along with the IFNβ luciferase reporter construct and TLR3 expression construct. Cells were stimulated with 25 mg/mL poly I:C directly added to the medium for 8 h. Luciferase activities correspond to the average of results from at least three independent experiments, and data are shown as means ± SE (**p<0.01, *p<0.05).

The expression of HBV Pol in the pAHC-Pol-transfected cell lines was confirmed by western blot as shown in Fig. 1A. The effect of HBV Pol on NF-κB activity in these cell lines was investigated using a luciferase reporter assay. The results in Fig. 1B show that HBV Pol significantly suppressed the activity of NF-κB elicited by TNF-α stimulation in HepG2 and Huh7 cells. As the proportion of pAHC-Pol increased, NF-κB activity was suppressed in a dose-dependent manner in 293T cells (Fig. 1C). Similar results were also observed for the HepG2 and Huh7 cell lines (data not shown). The results in Fig. 1D show that HBV Pol suppressed the activity of NF-κB after stimulation with TRAF2, TRAF6, IKKα, and IKKβ in HepG2 cells. To substantiate the above results in the context of viral life cycle, the impact of viral proteins on NF-κB signaling pathway was examined by using a viral replicon, which could lead to viral genome replication when transfected. Four mutants were made in which specific viral genes were inactivated, respectively: P (Pol)-null, E (HBeAg)-null, X-null and E/X (both HBeAg and X)-Null (Fig. S1). An increase in NF-κB luciferase activity by one of the HBV mutant constructs would point out that particular gene may contribute to the inhibition of NF-κB activity. To ensure that the physiological levels of viral proteins were attained, the amount of the three viral protein expression constructs for transfection were determined by cotransfection with the corresponding gene-null HBV replicon: for HBV Pol (see Fig. S2), for E protein and HBx (data not shown). Luciferase activity was monitored following transfection as described above. The data revealed that cells transfected with the HBV P-null replicon and E-Null constructs induced higher levels of NF-κB activity, whereas the other two replicons, X-Null and E/X-Null replicons inhibited NF-κB activity (Fig. 1E). Consistent with the above results, we did not observe the same inhibitory effect when the HepG2.2.15 virus-producing cells were assessed in comparison with wild-type HepG2 cells (Fig. 1F). Since previous work has reported the HBV X protein could stimulate transcription from a variety of promoter elements, including NF-κB [31], together with our observation that HBeAg also has an inhibitory effect to NF-κB, it is assumed that HBV Pol, E and X may regulate NF-κB activity coordinately at different stages during virus infection (see discussion). The results in Fig. 1G show a dose-related decrease of IFN promoter activity by HBV Pol expression when Cells were transfected with incremental dose of the HBV Pol and TLR3 expression constructs along with the reporter construct, followed by a treatment with poly I:C in the medium.

### HBV Pol Affects the Nuclear Translocation of NF-κB

**Figure 2:**
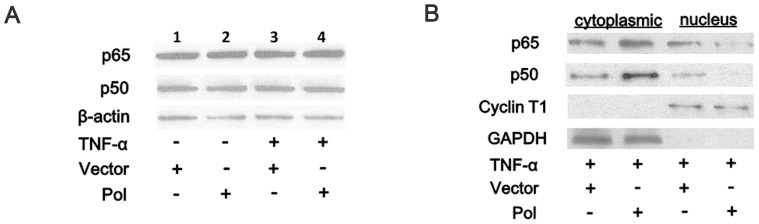
HBV Pol affects the nuclear translocation of NF-κB. 293(lanes 1 and 3) or with pAHC-Pol (lanes 2 and 4). Cells were examined at 24 h posttransfection and after stimulation or not with TNF-α (10 ng/ml) for 15 min (lanes 3 and 4). *A*. Cell extracts were prepared and analyzed by western blot using anti-p65, anti-p50, and anti-β-actin antibodies. *B*. Cytoplasmic and nuclear fractions were prepared as described in Materials and Methods. Both cytoplasmic and nuclear proteins were analyzed by SDS-PAGE and western blot with anti-p65 and anti-p50 antibodies to determine the localization of NF-κB subunits. An antibody against nuclear-specific cyclin T1 and an antibody against cytoplasmic-specific GAPDH were used as controls.

To investigate the molecular mechanism of NF-κB suppression in HBV polymerase-expressing cells, the total protein levels of NF-κB in HEK 293 T cells were examined by Western blot using antibodies specific for the subunits of NF-κB. As shown in Fig. 2A, the levels of the NF-κB subunits, p65 and p50, remained the same as in the control cells whether or not the cells were stimulated (comparing lane 1 with lane 2 and lane 3 with lane 4), suggesting that HBV Pol did not alter the expression of subunits of NF-κB. To determine the localization of NF-κB, cytoplasmic and nuclear fractions were prepared and examined by western blot using the same antibodies as above. The nuclear-specific antibody, cyclin-T1, was used as a control to exclude the possibility of cross contamination. We used a GAPDH control to ensure equal loading of the cytoplasmic fractions as well as of Cyclin-T1 in the nuclear fractions. As shown in Fig. 2B, HBV Pol inhibited the translocation of p50 and p65 to the nucleus, thereby increasing the amounts of cytoplasmic p50 and p65. These data demonstrated that HBV Pol prevented p50 and p65 from entering the nucleus, and hence, the viral protein inhibited the activity of NF-κB.

### HBV Pol Inhibits IKKα and IKKβ Protein Phosphorylation and IκBα Protein Degradation

**Figure 3:**
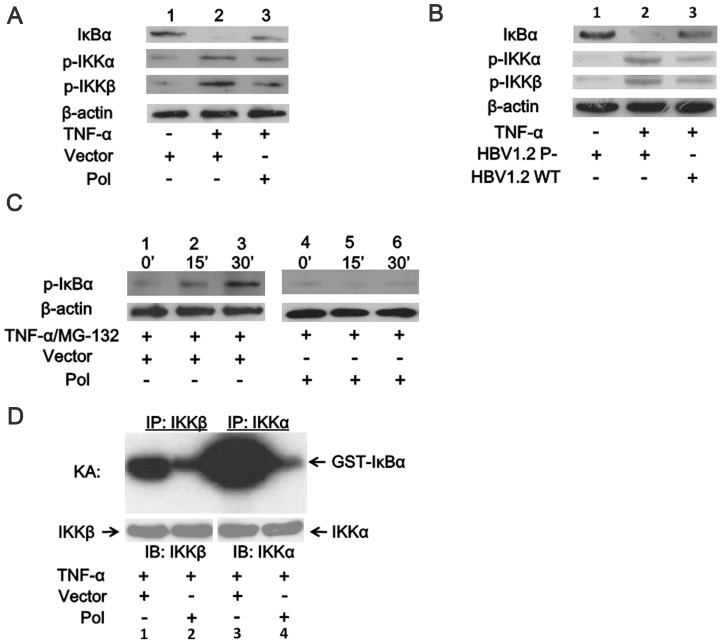
HBV Pol inhibits the activities of IKKα and IKKβ and the degradation of IκBα protein. 293(*A*.) and HepG2 cells (*B*.) were transfected with the plasmids as indicated. Cells were examined at 24 h posttransfection and after stimulation with TNF-α (10 ng/ml) or not for 15 min. Cell extracts were prepared, and IκBα, p-IKKα and p-IKKβ levels were analyzed by western blots. *C*. 293 T cells were transfected with the HBV Pol or the empty vector for 24 h, then the proteasome inhibitor, MG-132 (20 µM), was added for the indicated times, and cell extracts were prepared and assayed with a phosphoserine IκBα (P-IκBα) antibody. *D*. The cell extracts were immunoprecipitated by anti-IKKα or anti-IKKβ antibodies and further analyzed by an in vitro kinase assay (KA) using GST-IκBα (1–54) as the IKK substrate. The IKK protein level in each precipitate was also determined by western blot. β-actin was used as the control for the western blots.

The activation of NF-κB depends on the phosphorylation and polyubiquitination of IκBα, but phosphorylated IκBα (Ser 32/Ser 36) is an unstable intermediate due to its rapid proteasome-mediated degradation [32], [33]. Instead of detection of phosphorylated IκBα, we measured IκBα and phosphorylated IKKα/IKKβ levels by western blot to reveal whether HBV Pol suppresses NF-κB activation through prevention of activation of IKKα/IKKβ, which reduces the degradation of IκBα. We found that when HBV Pol was transfected into the 293 T cells, the amount of IκBα was substantially increased, while phosphorylated IKKα and IKKβ were down-regulated by the HBV Pol (Fig. 3A). Fig. 3B shows that more IκBα and lower levels of phosphorylated IKKα and IKKβ were detected in HepG2 cells transfected with the wild type HBV replicon, compared with that transfected with the P-null mutant, which substantiates the inhibitory effect of the HBV Pol on the IKK’s activity. We then pre-incubated the cells with the proteasome inhibitor, MG-132, to allow the accumulation of phosphorylated IκBα. As shown in Fig. 3C, the level of phosphorylated IκBα decreased in pAHC-Pol-transfected cells. We also performed an *in vitro* immunocomplex kinase assay to measure the effect of HBV Pol on TNF-induced IKK activation (Fig. 3D). For this assay, 293 T cells were transfected with pAHC-Pol or an empty vector control prior to TNF-α stimulation, and *in vitro* kinase assays were performed on the immunoprecipitated IKK complexes using an exogenous GST-IκBα fusion protein as the substrate. We found that HBV Pol inhibited the activation of IKK in response to TNF-α. Taken together, we concluded that HBV Pol inhibited IκBα protein degradation and blocked the release and translocation of the p65 and p50 subunits of NF-κB.

### HBV Pol Disrupts the Interaction of the Hsp90β/IKK Complex

**Figure 4:**
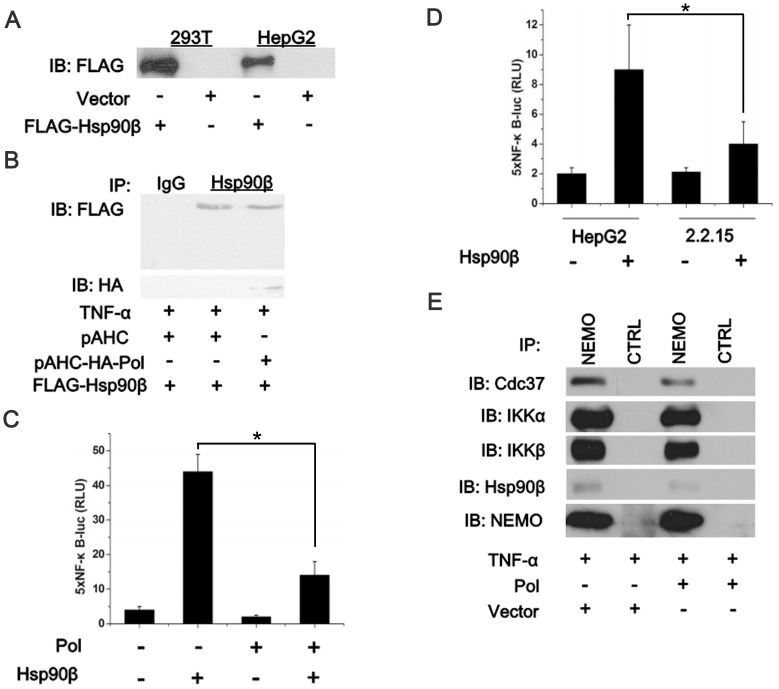
HBV Pol inhibits the activities of IKKs via interaction with Hsp90β. *A*. HEK 293 T and HepG2 cells were transfected separately with 0.8 µg of the pCMV-flag-2B empty vector as a control or pCMV-flag-Hsp90β expressing the Hsp90β protein. At 24 h posttransfection, cell extracts were prepared, and the expression of the plasmids was determined using an anti-Hsp90β antibody. *B*. HBV Pol interacts with Hsp90β. 293 T cells were transfected with either the empty vector (pAHC) or pAHC-Pol in parallel with pCMV-flag-Hsp90β. At 24 h posttransfection, cells were harvested and subjected to immunoprecipitation. One-twentieth of the total cell lysates used for immunoprecipitation was loaded as a positive control for Hsp90β. *C*. HepG2 cells were transfected with 5×pNF-κB-Luc, pTK-Renilla-Luc and other plasmids as indicated. At 24 h posttransfection, cells were harvested for analysis by luciferase assay. *D*. HepG2 and HepG2.2.15 cells were transfected with 5×pNF-κB-Luc reporting system and Hsp90β or the empty vector as control. At 24 h posttransfection, cells were harvested for analysis by luciferase assay. Luciferase activities correspond to the average of results from at least three independent experiments, and data are shown as means ± SE (*p<0.05). *E*. 293 T cells were transfected with pAHC-Pol or the empty vector (pAHC) as a control. At 24 h posttransfection and after stimulation with TNF-α (10 ng/ml) for 15 min, cell extracts were immunoprecipitated with a control IgG1 (CTRL) or anti-NEMO (NEMO) monoclonal antibody. The presence of Hsp90, Cdc37, IKKα/IKKβ, and NEMO in the immunocomplexes was determined by immunoblotting.

Recently, Hinz et al. reported that de novo synthesized IKKs depend on Hsp90-Cdc37 to achieve an enzymatically competent state. Upon stimulation by either receptor-mediated signaling or in response to DNA damage, mature IKKs again require Hsp90-Cdc37 following their T-loop phosphorylation to attain full catalytic activation [15]. Based on our above results, we hypothesized that HBV Pol may suppress the activity of IKKs via interaction with Hsp90β. To investigate whether HBV Pol performed its function in the NF-κB pathway via the Hsp90-Cdc37 complex, we cloned the Hsp90β coding segment into the pCMV-flag-2B vector to generate pCMV-flag-Hsp90β and verified its expression in Western blot (Fig. 4A). We then demonstrated that the Hsp90β could interact with HBV Pol by co-immunoprecipitation (Fig. 4B). Fig. 4C shows that the HBV Pol could inhibit NF-κB activity induced by Hsp90β. In addition, by using HepG2.2.15 cell line that stably expresses viral proteins and support HBV replication, we consistently found that NF-κB induced by Hsp90β was significantly diminished in HepG2.2.15 cells (Fig. 4D), indicating that the inhibitory function of HBV Pol was through Hsp90β and validating the impact of HBV Pol in a more physiological setting. Furthermore, we examined the possible role of HBV Pol in the association of IKK/NEMO with Cdc37/Hsp90. By using anti-NEMO immunoprecipitation, we isolated the IKK complexes from 293T cells that had been transfected with pAHC-Pol or empty vector after pretreatment with TNF-α. Subsequent immunoblotting analysis showed that HBV Pol suppressed the interaction between IKK/NEMO and Hsp90/Cdc37 (Fig. 4E).

### HBV Pol Suppresses NF-κB Activity in the Non-canonical NF-κB Pathway

**Figure 5:**
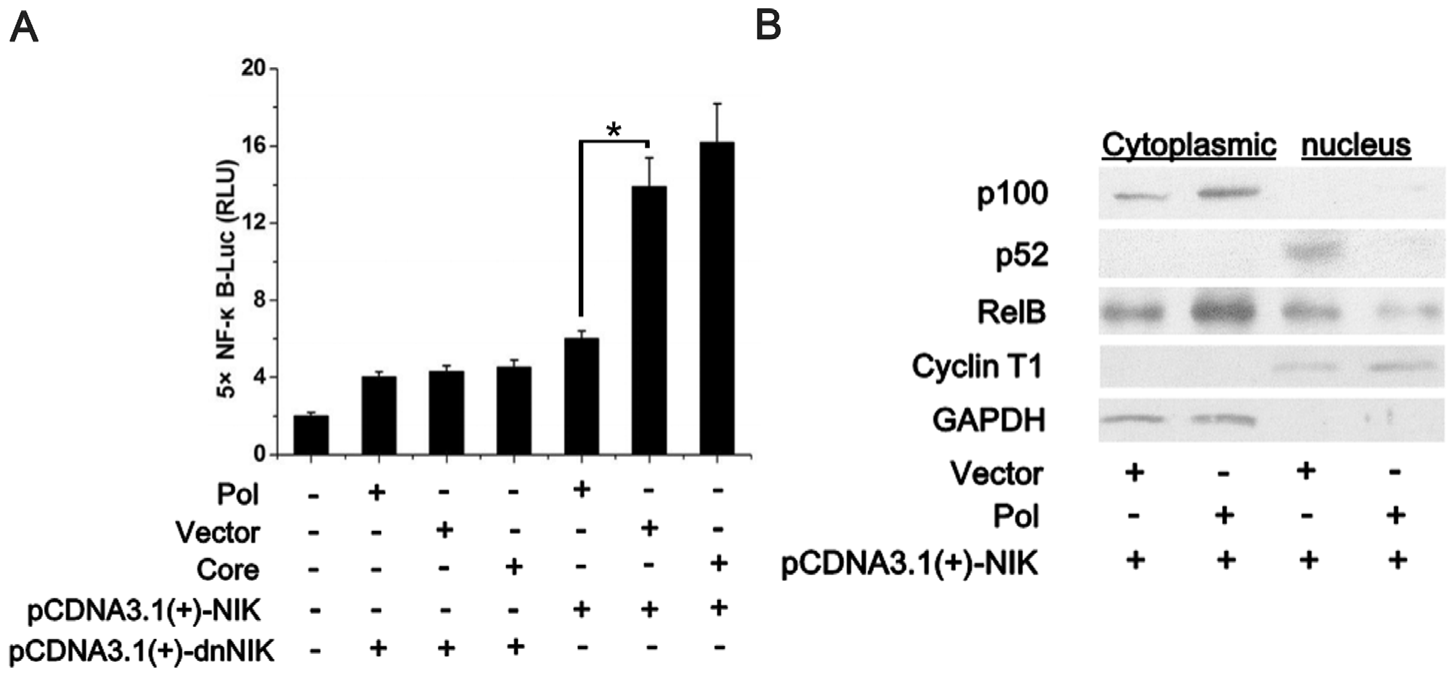
HBV Pol suppresses NF-κB activity in the non-canonical pathway. *A*. HepG2 cells were cotransfected with 100 ng of pcDNA3.1(+)-NIK or pcDNA3.1(+)-dnNIK, 100 ng of 5×pNF-κB-Luc, and 25 ng of pTK-Renilla-Luc together with 600 ng of pAHC-Pol or 600 ng of pAHC-Core and normalized to empty vector pAHC as indicated below the graph. At 24 h posttransfection, cells were examined for relative luciferase activities. Luciferase activities correspond to the average of results from at least three independent experiments, and data are shown as means ± SE (*p<0.05). *B*. 293 T cells were transfected with 0.1 µg of pcDNA3.1(+)-NIK and 0.6 µg of either the empty vector, pAHC, or pAHC-Pol as indicated below the graph. At 24 h posttransfection, cytoplasmic and nuclear fractions were prepared as described in Materials and Methods. Both cytoplasmic and nuclear proteins were analyzed by western blot using anti-p100/p52 and anti-Rel-B antibodies to determine the localization of NF-κB subunits. The nuclear-specific anti-cyclin T1 and cytoplasmic-specific anti-GAPDH antibodies were used as controls.

The non-canonical NF-κB pathway proceeds through proteasomal processing rather than via the degradation of p100 to p52, which liberates p52-containing NF-κB dimers that then drive gene transcription [26]. The non-canonical pathway depends only on the IKKα subunit, which functions by phosphorylating p100 and causing its inducible processing to p52. Based on the results above, we hypothesized that HBV Pol might be involved in the non-canonical NF-κB pathway. To examine whether HBV Pol affected the activity of NF-κB in the non-canonical NF-κB pathway, we performed an NF-κB luciferase assay. As shown in Fig. 5A, HBV Pol suppressed NF-κB activity elicited by NIK in the non-canonical NF-κB pathway. By detecting the localization and amount of p100/p52 (Fig. 5B), we confirmed that HBV Pol could inhibit the translocation of NF-κB in the non-canonical NF-κB pathway.

### HBV Pol Inhibits the NF-κB-mediated Transcription of Downstream Genes

**Figure 6:**
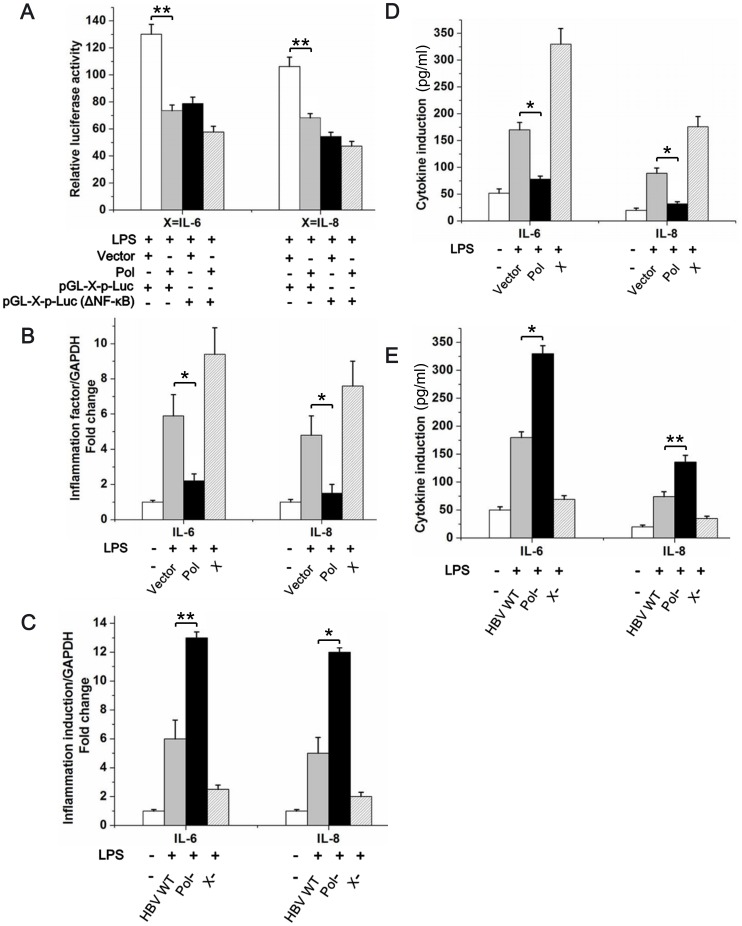
HBV Pol inhibits the expression of the NF-κB-mediated genes, IL-6 and IL-8. *A*. HepG2 cells were cotransfected with 0.1 µg of pGL3 X-p-Luc or pGL3 X-p-Luc (ΔNF-κB) (where X is IL-6 or IL-8) and 10 ng of pTK-Renilla-Luc along with 0.6 µg of pAHC or 0.6 µg of pAHC-Pol as indicated below the graph. At 36 h posttransfection, cells were treated with LPS (1 µg/ml) for 6 h and harvested for measurement of luciferase activity. *B*. and *C*. HepG2 cells were transfected with plasmids as indicated. At 24 h posttransfection, cells were stimulated with LPS (1 µg/ml) or left unstimulated for 6 h as indicated. GAPDH was used as a control. IL-6 and IL-8 mRNA was measured by RT-PCR and normalized by calculating the ratio of IL-6 or IL-8 mRNA to GAPDH. Data are expressed as means ± SE (**p<0.01, *p<0.05) to control levels. *D*. and *E*. HepG2 cells were transfected with the plasmids as indicated. At 24 h posttransfection, cells were stimulated with LPS (1 µg/ml) for 6 h. IL-6 and IL-8 in the culture supernatant of HepG2 cells were measured using the ELISA method. Data are expressed as means ± SE (**p<0.01, *p<0.05) to control levels. The results are representative of at least two independent experiments, each performed in triplicate.

IL-6 and IL-8 are both NF-κB-mediated genes, and NF-κB can bind directly to their promoters and regulate their expression. Given that HBV Pol inhibited NF-κB activity, we examined whether HBV Pol affected these genes. The results of a luciferase assay showed that HBV Pol affected the activity of the IL-6 and IL-8 promoters at NF-κB binding sites (Fig. 6A). We next measured the expression of IL-6 and IL-8 at the mRNA and protein levels. As shown in Fig. 6B, quantitative real-time PCR analysis revealed that LPS caused a remarkable increase in IL-6 and IL-8 mRNA compared with the levels in the control group, whereas the HepG2 cells transfected with pAHC-Pol treated with the same stimuli did not show this induction. Similar result was observed that when the HepG2 cells both induced by LPS, the P-Null construct caused a remarkable increase in IL-6 and IL-8 mRNA compared with the wild HBV1.2 (Fig. 6C). The protein levels of IL-6 and IL-8 were measured by ELISA, and Fig. 6D shows that the expression of both IL-6 and IL-8 decreased in pAHC-Pol-transfected HepG2 cells, and the expression of both IL-6 and IL-8 increased in Pol-Null-transfected HepG2 cells compared with the control group (Fig. 6E).

## Discussion

The activation of the NF-κB transcriptional program is a fundamental early step in immune activation, and mice deficient in different members of the NF-κB family are more susceptible to viral infection [34]. Therefore, interfering with the activation of NF-κB represents an exceptional strategy for a successful pathogen to counter multiple host innate defense processes by targeting a single host regulatory pathway. Many viruses disrupt innate immune responses and NF-κB through the use of multifunctional viral proteins that target specific aspects of the NF-κB pathway. However, this transcription factor has other functions that would favor viral infectivity, such as preventing apoptosis and promoting cell proliferation. It has been shown that different proteins from the same virus have evolved to manipulate the NF-κB pathway independently, and such proteins may be important at different stages of infection or in different cell types. For example, African swine fever virus (ASFV) regulates NF-κB activity in a biphasic manner, where virus-induced NF-κB activation is initially inhibited by the early viral protein, A238L, that acts as a degradation-resistant I-κB homologue [35]. However, once ASFV infection has progressed further, NF-κB activity is stimulated by the late viral protein, A224L, which has homology to cellular inhibitors of apoptosis (IAPs) and acts on both NF-κB and caspases to prevent apoptosis and prolong infection [36].

In case of HBV, previous data have indicated that the X protein, one of best studied proteins of HBV, can activate NF-κB through two distinct cytoplasmic pathways: by inducing phosphorylation and subsequent degradation of IκBα and by reducing the cytoplasmic levels of p105 [37]. Another evidence suggested that the HBV large surface proteins (LHBs) can also activate a variety of promoter elements, including PKC-dependent activation of AP-1 and NF-κB [38]. Intriguingly, our data presented that NF-κB activity was not enhanced upon TNF-α induction in HepG2.2.15 cell line compared to the parental HepG2 cells (Fig. 1F). It is tempting to speculate that certain viral components of HBV, either proteins or molecule, may inhibit NF-κB activation, which strengthened our finding that the HBV exerted the inhibition of NF-κB signaling by Pol. In our further study, we showed that HBV Pol suppressed the phosphorylation of IKKα and IKKβ, blocked IκBα degradation and inhibited NF-κB activation via interaction with Hsp90β. It remained to be learned how HBV abrogated the innate immune response, since IRF3 and NF-κB played important roles in the production of IFN-β, combined with the previous report that HBV Pol inhibited IFN-β induction by disrupting the interaction between IKKε and DDX3, the HBV Pol may downregulate IRF3 signaling pathway and NF-κB activity respectively and synergistically to counteract the IFN antiviral immune response.

Our data support another hypothesis, which postulates that HBV has developed strategies to suppress the initial antiviral response that is elicited by the innate immune system of the host. In accordance with this hypothesis, it has recently been demonstrated that HBV polymerase acts as a general inhibitor of IFN signaling and prevents IFN-inducible MyD88 expression by blocking the nuclear translocation of Stat1 [39].

In addition, other aspects of immune activation at the early stage of HBV infection have been unveiled. Within hours after infection, the NF-κB signaling pathway has been shown to be activated in both primary human hepatocytes (PHHs) and nonparenchymal cells (NPCs), which are mainly composed of Kupffer cells and liver sinusoidal endothelial cells (LSEC). IL-6 and other inflammatory cytokines (IL-8, tumor necrosis factor α, IL-1β) are then released, but no induction of type-1 interferon (i.e., IFN-β) expression or production has been observed. Intriguingly, the activation of NF-κB and the release of proinflammatory cytokines are transient after HBV infection and are not induced by newly synthesized virus. HBV replication tends to increase after 3–4 days following infection when the IL-6 level has already returned to baseline and remains stable afterward [40]. Our finding that HBV Pol dramatically reduced IL-6 and IL-8 levels by interfering with the NF-κB signaling pathway may partially explain why the virus induces little detectable innate immune responses during early infection. It has been shown that the HBV X protein can induce several cytokines and chemokines, including IL-6, IL-8, and TNF-α [41]–[43]. Based on our observations, we induced that on the early stage of the infection of HBV, X protein was released firstly and Pol was still enclosed by the nucleocapsid, then the NF-κB signaling pathway was activated. While with the transcription of the HBV genome and translation of the novel viral proteins including HBV Pol, the newly synthesized HBV virus would exert both inhibitive and induced effects to the NF-κB signaling pathway, NF-κB activity would reduce and return to the baseline level.

In conclusion, our results indicate that the HBV polymerase can counteract the antiviral response of the local innate immune system of the liver by antagonizing NF-κB-mediated induction of proinflammatory cytokines. This is relevant for our understanding of the interaction between HBV and the innate immune system and may in part explain why HBV appears to act as a “stealth virus” in the initial phase of infection.

## Supporting Information

Figure S1 **The Map of HBV replicon constructs used in this study.** WT represents the 1.2 mer over-the-genome length HBV replicon construct. Three ORFs are drawn on the pregenomic RNA, but S ORF is omitted for clarity. Three mutant replicon constructs including the P-null, E-null, and X-null and E/X-Null constructs are drawn with the introduced stop codons denoted by dots. The ORF with dashed line denotes the inactivated ORF.
(TIF)

Figure S2 **Real time PCR analysis of viral DNA isolated from cytoplasmic capsids.** Cells were transfected either with the wild-type HBV replicon or the P-null replicon along with an increasing amount of the Pol expression construct: 200, 400, and 600 ng per 24-well plate respectively. Viral DNAs isolated from cytoplasmic capsids were analyzed by real time PCR analysis. E-Null, X-Null, E/X-Null replicons were transfected as controls.
(TIF)
