# Supplementary figures and images for: Hepatitis B Virus Polymerase Suppresses NF-κB Signaling by Inhibiting the Activity of IKKs via Interaction with Hsp90β
Source: PLoS One. 2014 Mar 11;9(3):e91658. doi: 10.1371/journal.pone.0091658 (PMC3950214; doi:10.1371/journal.pone.0091658)

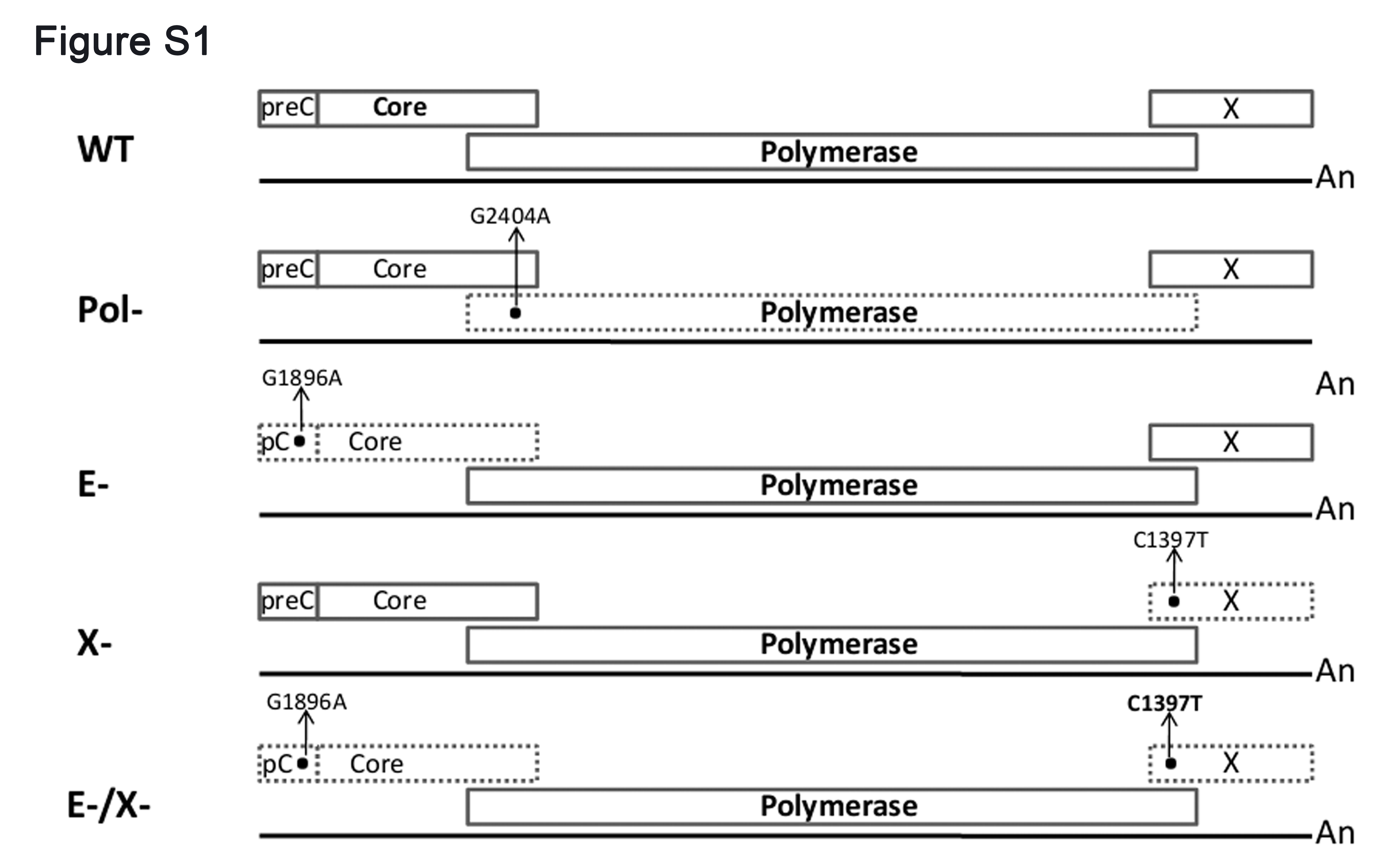

Supplement: Figure S1 — The Map of HBV replicon constructs used in this study. WT represents the 1.2 mer over-the-genome length HBV replicon construct. Three ORFs are drawn on the pregenomic RNA, but S ORF is omitted for clarity. Three mutant replicon constructs including the P-null, E-null, and X-null and E/X-Null constructs are drawn with the introduced stop codons denoted by dots. The ORF with dashed line denotes the inactivated ORF. (TIF) [file pone.0091658.s001.tif]

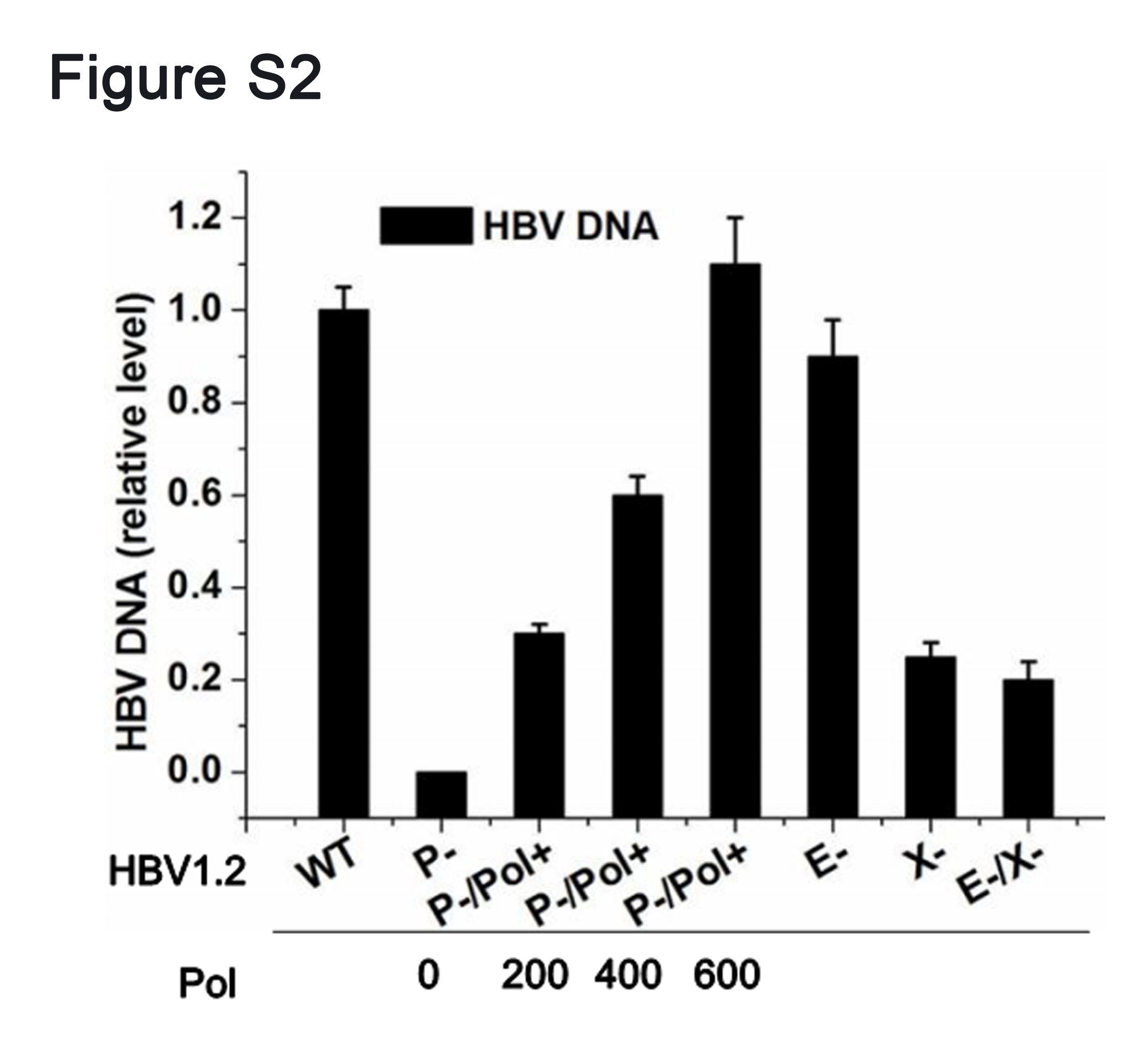

Supplement: Figure S2 — Real time PCR analysis of viral DNA isolated from cytoplasmic capsids. Cells were transfected either with the wild-type HBV replicon or the P-null replicon along with an increasing amount of the Pol expression construct: 200, 400, and 600 ng per 24-well plate respectively. Viral DNAs isolated from cytoplasmic capsids were analyzed by real time PCR analysis. E-Null, X-Null, E/X-Null replicons were transfected as controls. (TIF) [file pone.0091658.s002.tif]
